# A novel prognostic related lncRNA signature associated with amino acid metabolism in glioma

**DOI:** 10.3389/fimmu.2023.1014378

**Published:** 2023-04-11

**Authors:** Qiang Lei, Bo Yuan, Kun Liu, Li Peng, Zhiwei Xia

**Affiliations:** ^1^ Department of Neurology, The Second Xiangya Hospital, Central South University, Changsha, Hunan, China; ^2^ Department of Cerebrovascular Surgery, The Second People’s Hospital of Hunan Province, The Hospital of Hunan University of Chinese Medicine, Changsha, Hunan, China; ^3^ Department of Ophthalmology, Central South University Xiangya School of Medicine Affiliated Haikou Hospital, Haikou, Hainan, China; ^4^ Department of Ophthalmology, The Second Xiangya Hospital, Central South University, Changsha, Hunan, China; ^5^ Department of Neurology, Hunan Aerospace Hospital, Changsha, Hunan, China

**Keywords:** glioma, lncRNA, amino acid, prognostic, target

## Abstract

**Background:**

Glioma is one of the deadliest malignant brain tumors in adults, which is highly invasive and has a poor prognosis, and long non-coding RNAs (lncRNAs) have key roles in the progression of glioma. Amino acid metabolism reprogramming is an emerging hallmark in cancer. However, the diverse amino acid metabolism programs and prognostic value remain unclear during glioma progression. Thus, we aim to find potential amino-related prognostic glioma hub genes, elaborate and verify their functions, and explore further their impact on glioma.

**Methods:**

Glioblastoma (GBM) and low-grade glioma (LGG) patients’ data were downloaded from TCGA and CCGA datasets. LncRNAs associated with amino acid metabolism were discriminated against *via* correlation analysis. LASSO analysis and Cox regression analysis were conducted to identify lncRNAs related to prognosis. GSVA and GSEA were performed to predict the potential biological functions of lncRNA. Somatic mutation data and CNV data were further built to demonstrate genomic alterations and the correlation between risk scores. Human glioma cell lines U251 and U87-MG were used for further validation *in vitro* experiments.

**Results:**

There were eight amino-related lncRNAs in total with a high prognostic value that were identified *via* Cox regression and LASSO regression analyses. The high risk-score group presented a significantly poorer prognosis compared with the low risk-score group, with more clinicopathological features and characteristic genomic aberrations. Our results provided new insights into biological functions in the above signature lncRNAs, which participate in the amino acid metabolism of glioma. LINC01561 is one of the eight identified lncRNAs, which was adopted for further verification. In *in vitro* experiments, siRNA-mediated LINC01561 silencing suppresses glioma cells’ viability, migration, and proliferation.

**Conclusion:**

Novel amino-related lncRNAs associated with the survival of glioma patients were identified, and a lncRNA signature can predict glioma prognosis and therapy response, which possibly has vital roles in glioma. Meanwhile, it emphasized the importance of amino acid metabolism in glioma, particularly in providing deeper research at the molecular level.

## 1 Introduction

Gliomas are among the most severe and common primary human brain malignancies with a dismal prognosis and a very low 5-year survival rate, which are characterized by an immunosuppressive microenvironment (1–3). Currently, the clinical treatment includes surgery, chemotherapy, radiotherapy, targeted therapy, and immunotherapy in gliomas (4, 5). However, patients with gliomas remain to have a poor prognosis, especially in high-grade gliomas (6). Hence, further exploring the mechanism of glioma is of great significance for finding new targets for the treatment of glioma.

The microenvironment is recognized as playing a vital role in the development and progression of tumors (7, 8). In recent years, it has been recognized as an emerging hallmark of cancer (9, 10), which plays a major role in cancer occurrence and development. Metabolism reprogramming has been recognized as having a critical role in both cancer progression and effective immune responses in the tumor microenvironment (11). Metabolism must be altered to meet the rapid biosynthetic demands for growing tumors in metabolism proliferating cancer (12). As with sugar metabolism, amino acid metabolism is also an ordered process, which is important for the maintenance of cellular homeostasis (13). Amino acid transport is a vital aspect of amino acid metabolism. Amino acids and their metabolites play a critical role in metabolism and are essential to life (14). Cancer cells need large amounts of energy and compounds to meet the requirements of metabolic reprogramming. Amino acid metabolism affects the prognosis of tumor (15). The uptake and metabolism of amino acids are increased in cancer cells *via* upregulation of specific amino acid transporters (16). Amino acids promote the proliferation and survival of cancer cells in the context of genotoxic, oxidative damage, nutritional change, and stress (11, 16). Currently, a new focus on the amino acid metabolism of glioma is considered to play a major role in glioma. Therefore, better knowledge of amino acid metabolism in terms of cancer is strongly warranted.

Long non-coding RNA (lncRNA) has been reported to be involved in multiple physiological and pathological processes and has shown an essential role among them (17). Glioblastoma cells increase under conditions of hypoxic stress (18, 19). Amino acid metabolism-related risk signatures can be used to predict the prognosis for glioma (19). In addition, studies have demonstrated that lncRNA has shown a crucial role in the proliferation, progression, invasion, and prognosis of gliomas (20–22). Therefore, research about new lncRNAs as novel biomarkers will provide deeper insights into the progression and prognosis of glioma with the purpose of strengthening the management of the disease.

Previous studies have indicated that amino-related lncRNAs are involved in the prognosis of malignant tumor (23, 24). However, the role of amino acid‐related gene sets remains unclear in glioma. Herein, we collected data from The Cancer Genome Atlas (TCGA) and Chinese Glioma Genome Atlas (CGGA) databases by using multiple algorithms to investigate the prognostic value of amino-related lncRNAs and correlation in the microenvironment of glioma.

## 2 Methods

### 2.1 Data resources

Human gene expression profiles and corresponding clinical information on glioma were downloaded from TCGA dataset (http://cancergenome.nih.gov) and the CGGA dataset (http://www.cgga.org.cn/). TCGA dataset was regarded as the training set, the CGGA dataset as the validation set. Log2(tpm+0.001) was used to normalize the data of gene expression. We collected 672 samples from TCGA dataset and 322 samples from the CGGA dataset. Somatic mutation and copy number variation (CNV) data were obtained from the dataset of TCGA.

### 2.2 Screening for prognostic lncRNAs associated with amino acids

**Figure 1:**
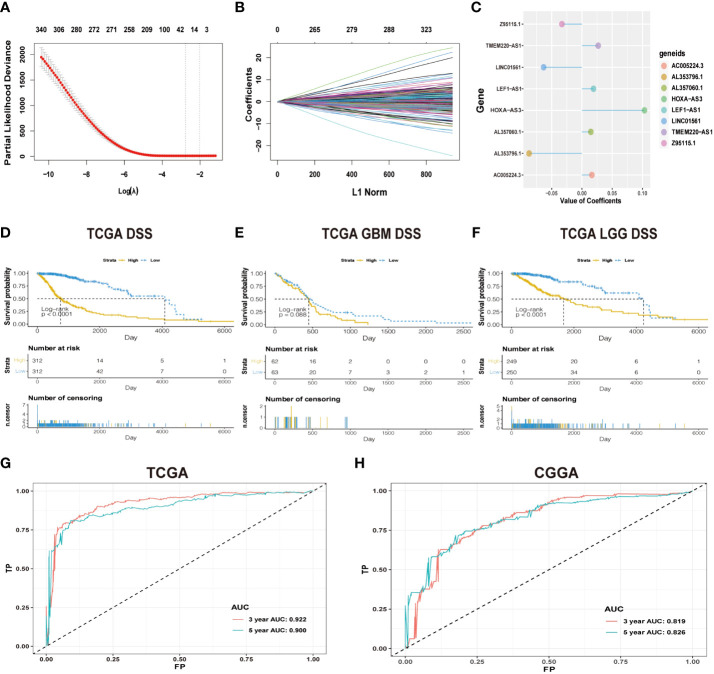
**(A)** Cross-validation in LASSO regression; dashed lines indicate the best-fit log (λ) value. **(B)** LASSO coefficients of the lncRNAs that have independent prognostic value. **(C)** Coefficient profie of the eight signature lncRNAs. **(D-F)** Difference in K-M curves of the DSS between high- and low-risk groups in the dataset of TCGA. **(G)** The ROC for predicting the survival at 3 and 5 years in TCGA dataset. The AUC of the risk score model showed good accuracy in the dataset of TCGA. **(H)** ROC for predicting the survival at 3 and 5 years in the dataset of CGGA.

To extract the amino-related lncRNA, gene set variation analysis (GSVA) was implemented using the GSVA R package. The amino-related gene sets were extracted from the Molecular Signatures Database (MSigDB) (https://www.gsea-msigdb.org/gsea/msigdb/i). A correlation analysis was conducted using the limma package of R statistical software. Amino-related lncRNAs were characterized *via* correlation analysis on the basis of Gene Ontology (GO) information of the target amino-related gene sets. Then, a univariate Cox regression analysis was performed to analyze all the lncRNAs related to the glioma patients’ overall survival, and a multivariable Cox regression analysis was used to further select and identify lncRNAs that exhibited independent prognostic value. LASSO regression analysis was used in the construction of the lncRNAs with prognostic gene signatures. Then, we obtained a set of prognostic lncRNAs and regression coefficients (β) (**Figure 1**).

### 2.3 LncRNAs used for consensus clustering

Consensus clustering was performed to identify glioma subgroups (GBM and LGG) and cluster amino-related genes from TCGA dataset using the ConsensusClusterPlus R package. Permuting clustering runs by changing the category number k = 2 to k = 10. Based on a relatively high consistency within the clusters, cumulative distribution function (CDF) and a relatively small incremental change in area under the CDF curve were used to determine the optimal number of clusters k. Survival analyses were conducted with R statistical software and packages.

### 2.4 Genomic alteration cluster

Somatic mutation analysis and copy number variation (CNV) were utilized to determine whether the risk score levels were related to specific genomic characteristics in gliomas *via* TCGA dataset. The somatic mutation data were analyzed with the “maftools” R package, and the genes with the most frequent somatic mutations were screened and presented. The enrichment of genomic events was determined by GSITIC analysis through an online analysis platform (https://www.genepattern.org).

### 2.5 Constructing a prognostic risk score model based on the clinical features and risk score

Univariate survival analyses were carried out by using the Cox proportional hazards regression package for the risk score and clinical features (age, gender, risk, IDH status, grade, 1p/19q) with a cutoff of p-value less than 0.05. Then, the multivariate Cox regression model was built based on the selected features, and visualization was achieved through a nomogram chart with the Regplot package. The area under the curve (AUC) value of the receiver operating characteristic (ROC) curve was established as an effective risk model to evaluate the prognosis of high-risk and low-risk patients. Both the calibration curve and AUC were carried out to evaluate the risk model.

### 2.6 Gene set variation analysis

Gene set variation analysis (GSVA) was performed using the ClusterProfiler package of R to calculate the enrichment analysis within TCGA and CGGA samples. The correlation between the risk score and GO terms was performed based on the significant GO terms of biological processes identified (p < 0.05), and a high correlation coefficient was selected.

### 2.7 Predicting the response to immunotherapy

The TIDE algorithm (http://tide.dfci.harvard.edu/) and the submap algorithm on the GenePattern website (https://cloud.genepattern.org/gp) are used to predict the likelihood of response to immune checkpoint blockade for individual samples and subtypes.

### 2.8 Cell culture, treatments, and siRNA transfection

Human glioma cells (U251 and U87) were purchased from Procell Life Science & Technology Company (Hubei, China). The logarithmic growth phase cells were grouped into the following groups: control group, siRNA-negative control (NC) group, and LINC01561-siRNA group. Cells were treated with 5 µl siRNA, and Lipofectamine 2000 was diluted in Opti-MEM medium for 5 min. They were then mixed and incubated at room temperature for 20 min. The composite was then added to the cell culture plate. Following transfection for 48 h, the cells were collected for the subsequent experiments.

### 2.9 RT-qPCR assay

RT-qPCR assay was performed. The primers of β-actin (F: ACCCTGAAGTACCCCATCGAG, R: AGCACAGCCTGGATAGCAAC) and LINC01561 (F:CCAGGAGGAGCAGAGAAAGC, R: CCCAGCTGCTGTCTGGTTTA) were designed using Primer Premier 5.0. The total RNAs were extracted and then reversely transcribed into cDNA by HiScript Q RT SuperMix for RT-qPCR. The expression levels of β-actin and LINC01561 were quantified and calculated with the method of 2-ΔΔCT. The reaction conditions were as follows: 95°C for 10 min, then 95°C for 15 s, and 60°C for 30 s, for a total of 40 cycles.

### 2.10 Validation *in vitro* cell experiments

Cell viability was carried out by using a cell counting kit-8 (CCK-8) assay. Cell migration was measured by the Transwell migration assay.

### 2.11 Colony formation assay

Colony forming ability was assessed by a colony formation assay. Cell proliferation was evaluated and measured by using the 5-ethynyl-2′-deoxyuridine (EdU) assay kit (RiboBio, China). Each group has three biological replicates. Detailed vitro experimental protocol can be found in the supplemental material (**Table S1**).

### 2.12 Statistical analysis

Statistical calculations were performed using R statistical analysis package (version 3.5.3). One-way ANOVA followed by the Tukey posttest was used to identify the differences among groups, respectively. Correlations between categorical variables were assessed using chi-square tests. Overall survival analysis was evaluated using the Kaplan–Meier method, followed by Cox regression analysis. The ClusterProfiler package was performed to measure the enrichment analysis in TCGA and CGGA samples. Both the somatic mutations and CNA data were obtained through TCGA database. A comparison was accepted to be statistically significant when a p value was <0.05.

## 3 Results

### 3.1 Identification of prognostic lncRNAs related to amino acids

**Figure 2:**
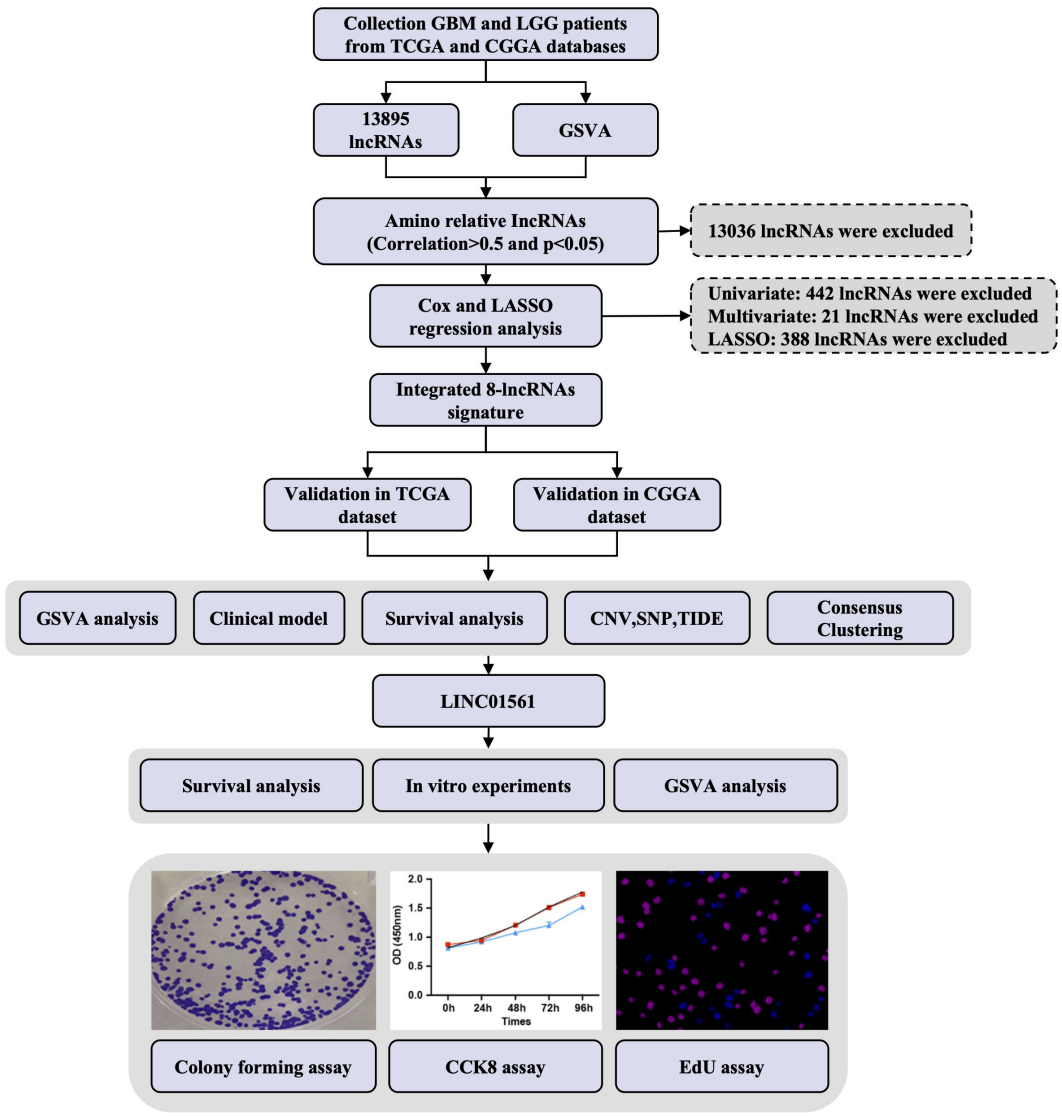
Study design flowchart.

The workflow diagram of this study is illustrated in **Figure 2**. In total, in TCGA and CCGA datasets, 13,895 lncRNAs were extracted *via* intersecting the lncRNAs. Univariate and multivariate Cox regression analyses were carried out to find information on the lncRNA expression level of patients between the lncRNA and overall survival (OS) *via* the survival R package. There were 417 and 396 lncRNAs with significant prognostic potential screened out by univariate and multivariate Cox regression analysis (p < 0.05), respectively. Meanwhile, LASSO regression is an effective method for high-dimensional data and predictors. Then, in both TCGA and CCGA datasets, there were eight considered amino-related lncRNAs regarded with independent prognostic values identified *via* LASSO regression (**Figures 1A, B**), and the coefficient profile of the eight signature lncRNAs is illustrated in **Figure 1C**. They were AL357060.1, HOXA-AS3, LINC01561, Z95115.1, AL353796.1, LEF1-AS1, AC005224.3, and TMEM220-AS1. Then, a prognostic amino-related lncRNA signature was built (**Figures 1D–F**). The sensitivity and specificity of the eight-lncRNA prognostic model were determined by measuring the area under the receiver operating characteristic (AUC) and ROC between 3-year survival and 5-year survival. The AUC was 0.922 at 3-year survival and 0.900 at 5-year survival in TCGA dataset. The AUC was 0.819 at 3-year survival and 0.826 at 5-year survival in the CGGA dataset (**Figures 1G, H**). It can be seen that the AUC of the ROC and risk scoring models predicting the survival rates at 3 and 5 years show good accuracy in the two datasets.

### 3.2 Consensus cluster analyses of the 8 lncRNA expression

**Figure 3:**
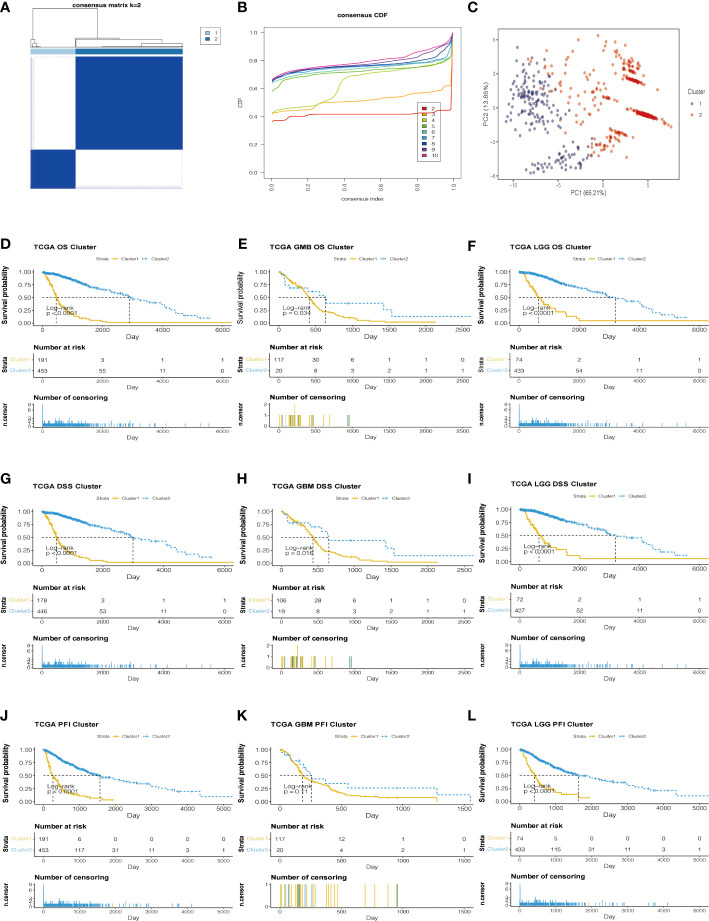
Consensus Cluster analyses of the 8 lncRNA expressions in the TCGA dataset. **(A)** Clustering stability for k=2 to 10. **(B)** The incremental change is small under the CDF curve area when k = 2. **(C)** The consensus clustering of gliomas was clustered into two clusters in principal component analysis (PCA). **(D–L)** The K-M plots of the difference in OS, DSS, and PFI between cluster 1 and cluster 2 in TCGA datasets.

Based on the analysis of TCGA data, genes related to prognosis were identified. Consensus clustering analysis was applied for grouping the samples of glioma patients. With the expression similarity based on the dataset of TCGA, clustering stability rises from k = 2 to k = 10. The incremental change is small in the area under the CDF curve when k = 2 (**Figures 3A, B**). Hence, k = 2 was considered an optimal choice. Consensus clustering of gliomas was clustered into two clusters as follows: cluster 1 and cluster 2. Moreover, cluster 1 was associated with a worse poor prognosis. Meanwhile, cluster 1 was worse than cluster 2 in overall survival (OS), disease-specific survival (DSS), and progression-free interval (PFI) (**Figures 3D–L**). Based on the eight prognostic-related lncRNAs, the results of the principal component analysis of the two clusters showed that there was an obvious separation between the survival probability distribution and PCI distribution in TCGA and CGGA datasets (**Figures 3C**, **S3C**).

### 3.3 Establish and validate the risk score model

According to the median risk score, patients were assigned into high-risk and low-risk groups. The results showed that high-risk patients had a significantly worse prognosis than the low-risk group in both TCGA dataset and CGGA dataset (p < 0.05, **Figures 1**, **S1**). To further evaluate the clinical endpoints derived from the two risk groups, DSS, OS, and PFI were used to compare the survival results of the patients in TCGA dataset. OS was used to compare the survival results of the patients in the CGGA dataset. Univariate analysis shows that when we use DSS, OS, and PFI as clinical endpoints, the high-risk group showed a worse prognosis than the low-risk group in TCGA dataset (**Figures 1D–F**, **S1A–F**) and OS in the CGGA dataset (**Figures S1G–I**).

### 3.4 Risk models of different subgroups of gliomas

To explore the survival status of different subgroups of gliomas, single-factor Cox regression analysis was performed on the 1p/19q status, gender, age, pathological grade, IDH type, MGMT methylation status, cancer type, and other factors in TCGA database. In addition to the gender of patients, other subtypes are associated with prognosis. Patients with MGMT methylation had lower risk scores (**Figures S2A–I**). The patient’s risk is related to age. Patients older than 45 years of age have a higher risk than patients younger than 45 years. 1p/19q no deletion and IDH wild type have higher risk scores with a poor prognosis. As the pathological grade of glioma increases, the risk score also increases and has a poor prognosis. These results demonstrate that clinical features were significantly associated with the risk score of the model, and a significant positive correlation between high-risk scores and dangerous clinical features was observed (**Table 1**).

**Table 1:** Clinical baseline characteristics between high- and low-risk groups in TCGA dataset.

Characteristic	N	High (N = 322)	Low (N = 323)	p-value
Age	645			<0.001
		96 (30%)	222 (69%)	
		226 (70%)	101 (31%)	
Gender	645			>0.9
Female		136 (42%)	136 (42%)	
Male		186 (58%)	187 (58%)	
Cancer	645			<0.001
GBM		136 (42%)	1 (0.3%)	
LGG		186 (58%)	322 (99.7%)	
IDH	638			<0.001
Mutant		113 (36%)	308 (95.7%)	
WT		203 (64%)	14 (4.3%)	
1p/19q	641			<0.001
Codel		30 (9.4%)	138 (43%)	
Non-codel		289 (90.6%)	184 (57%)	
MGMT	614			<0.001
Methylated		171 (59%)	292 (90.4%)	
Unmethylated		120 (41%)	31 (9.6%)	

### 3.5 Correlation between genomic alterations and the glioma risk model

**Figure 4:**
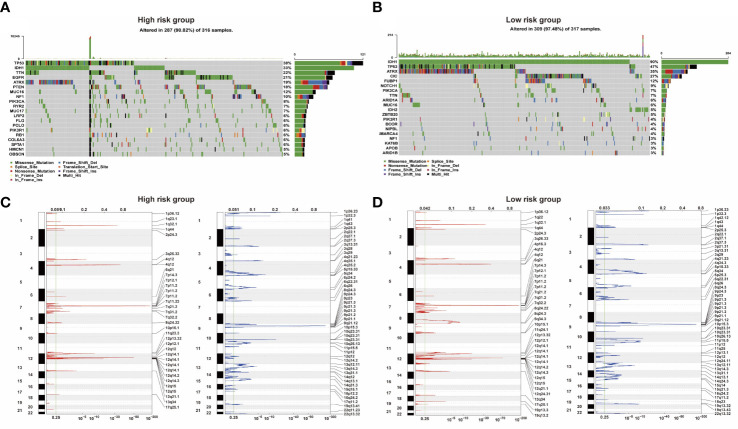
**(A, B)** Two groups of somatic mutation profiles. **(C, D)** Deleted and amplified chromosomal regions were presented in blue and red in high- and low-risk groups.

Analysis of somatic mutation and CNV showed that there were significant differences between the low-risk and high-risk groups. Compared with the overall population, high-risk groups of patients had more gene mutations on chromosomes 7 and 10 and fewer gene mutations on chromosome 1. 9p is prone to copy number deletion gene mutation, and 1q, 4q, 7p, and 12q are prone to copy number amplification gene mutation in the high-risk group (**Figures 4C, D**). IDH1, TP53, ATRX, and CIC were the main variant genes in the low-risk group, whereas TP53, IDH1, ATRX, and EGFR were the main CNV genes in the high-risk group. Patients with low-risk scores had significantly higher mutation frequencies of IDH1 and TP53 than high-risk score patients (IDH1, 90% vs. 33%; TP53, 47% vs. 38%) (**Figures 4A, B**). The risk of IDH1 missense mutations is significantly reduced within the high-risk group. IDH1 missense mutations are related to low-grade gliomas and may bring patients better prognoses. The variation of CIC (27%) mainly occurred in the low-risk group. PTEN (18%) mutations mainly occur in high-risk groups. Studies have shown that PTEN deficiency can cause PAX7 to be up-regulated, which in turn promotes the carcinogenic transformation of NSC, which may be associated with a poor prognosis.

### 3.6 Functional assays of the selected prognostic lncRNAs

**Figure 5:**
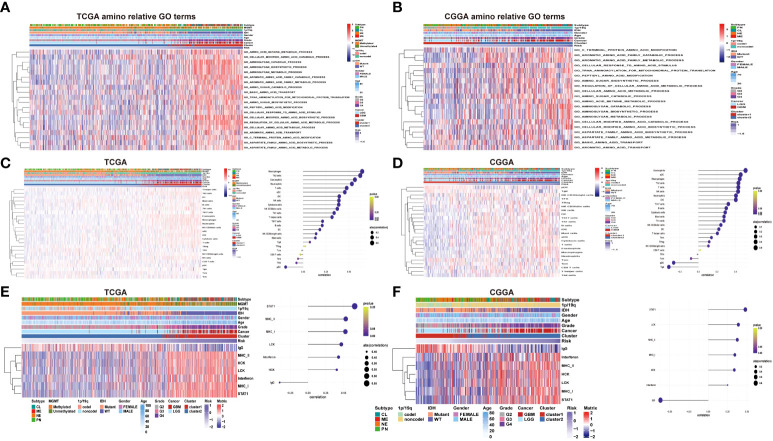
**(A–B)** Heatmaps of the identified amino acid metabolism functions, clinicopathological characteristics, and risk score in the TCGA and CGGA datasets. **(C–D)** Heatmap of immune infiltrating cells in different levels of the risk score in TCGA and CGGA datasets. **(E–F)** Heatmap of inflammatory signature genes under different risk score levels in both the TCGA and CGGA datasets.

To further verify the potential function and target gene of the eight prognostic lncRNAs, GSVA was used to perform with TCGA and CGGA data. It can be seen that the representative GO terminology is significantly related to the prognostic lncRNA in TCGA dataset. Samples from the high-risk group were enriched with more biological processes related to amino acid metabolism compared with the low-risk group. In both datasets of TCGA and CGGA, the 20 most significant biological processes of amino metabolism correlated with risk score were identified by correlation analysis of both TCGA and CGGA datasets, respectively (**Figure 5**). The most enriched functions of lncRNAs included regulation of cellular amino acid metabolism processes, aromatic amino acid family metabolism, cell-modified amino acid biosynthesis, aromatic amino acid family metabolic process, and amino sugar metabolism. Among them, cell-modified amino acid biosynthesis, aromatic amino acid family metabolic processes, and amino sugar metabolic processes are strongly and positively correlated. The lncRNA risk model is involved in the regulation of the tumor microenvironment, directly or indirectly promoting tumor cell growth, tumor blood vessel formation, migration and invasion, and inhibiting cell apoptosis.

### 3.7 Prognostic nomogram assessment of overall survival prediction

**Figure 6:**
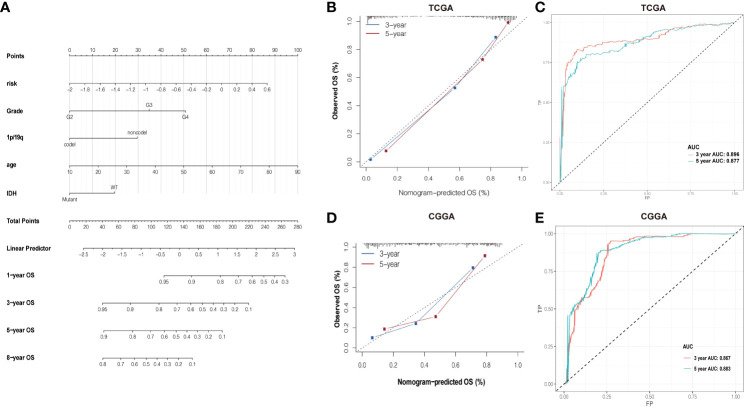
**(A)** Nomogram of summing the points for each variable. **(B, D)** The difference in K-M plots of the nomogram predicted OS between high- and lowrisk groups in both TCGA and CGGA datasets. **(C, E)** Nomogram of ROC curves and AUC values in both TCGA and CGGA datasets.

Based on the association between clinicopathological characteristics and risk score, a nomogram was conducted based on age, glioma risk, 1p/19q, IDH mutation, and glioma grades to predict the survival rate of patients (**Figure 6A**). The predicted 3- and 5-year survival was agreed strongly with the actual rates in TCGA and CGGA datasets (**Figures 6B, D**). The AUC and ROC were performed to compare the survival rate at 3 and 5 years and the risk score model showed a good consistency (**Figures 6C–E**).

### 3.8 LINC01561 is a prognosis-related biomarker of gliomas correlated with immune infiltration

**Figure 7:**
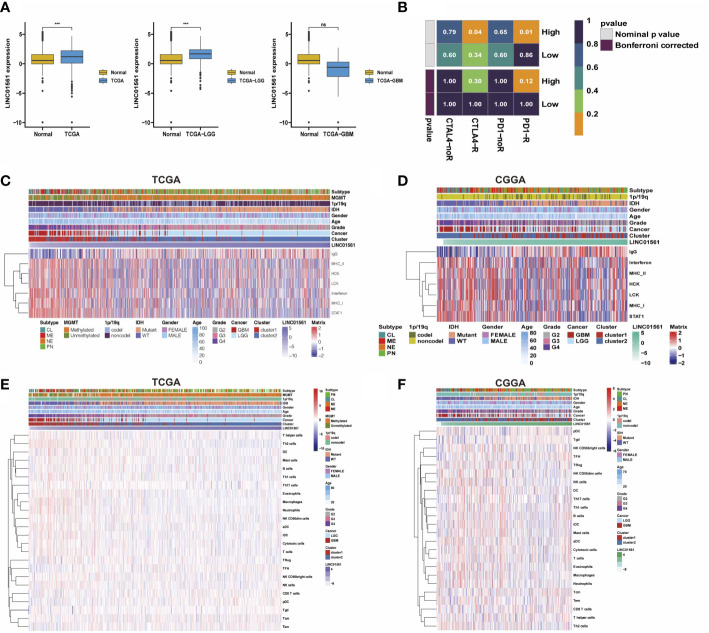
**(A)** Comparison of LINC01561 expression in different groups. **(B)** The differences of the risk score were compared using submap analysis in the anti-PD-1 response and anti-cytotoxic T lymphocyte-associated antigen-4 (CTLA-4) response. **(C-F)** Correlograms between immune cell infiltration, inflammation activities, and LINC01561 expression in TCGA dataset and the CGGA dataset. ***p < 0.001.

LINC01561 has been reported in other cancers, but its effect on glioma has not been reported. Hence, LINC01561 was chosen for further analysis. We compared GBM and LGG samples in TCGA dataset with normal samples in the GTEx dataset in the GEPIA online database (http://gepia.cancer-pku.cn). The expression of LINC01561 in the tumor samples is higher than that in the normal samples, but significant differences were found only in the LGG samples (**Figure 7A**).

In addition to the lncRNA signature, the prognostic value in gliomas was also further confirmed. GSVA results also indicated that LINC01561 may be involved in regulating the prognosis of gliomas. The tumor immune microenvironment is considered closely associated with cancer initiation, prognosis, and response to immunotherapy (25). Submap analysis manifested that the high risk-score group is more sensitive to anti–PD-1 therapy and CTLA4 therapy (nominal p value <0.05, **Figure 7B**), suggesting that it may benefit from immunotherapy. Moreover, the correlation between LINC01561 and immune activity was explored. The correlation analysis showed that the infiltration of pDC, TFH, Tem, CD8T cells, and Tgd were positively associated with the expression of LINC01561, whereas TH17 cells, NK CD56bright cells, B cells, Treg, mast cells, DC, Th1 cells, NK cells, T helper cells, cytotoxic cells, NK CD56dim cells, iDC, eosinophils, aDC, T cells, neutrophages, macrophages, and Th2 cells were negatively associated. On the level of the inflammatory response, the expression of LINC01561 was positively correlated with IgG, MHC-II, STAT1, LCK, MHC-I, HCK, and interferon. These results hinted that LINC01561 was involved in regulating the immune microenvironment of gliomas (**Figures 7C–F**).

### 3.9 LINC01561 affected glioma cell viability, migration, and proliferation

**Figure 8:**
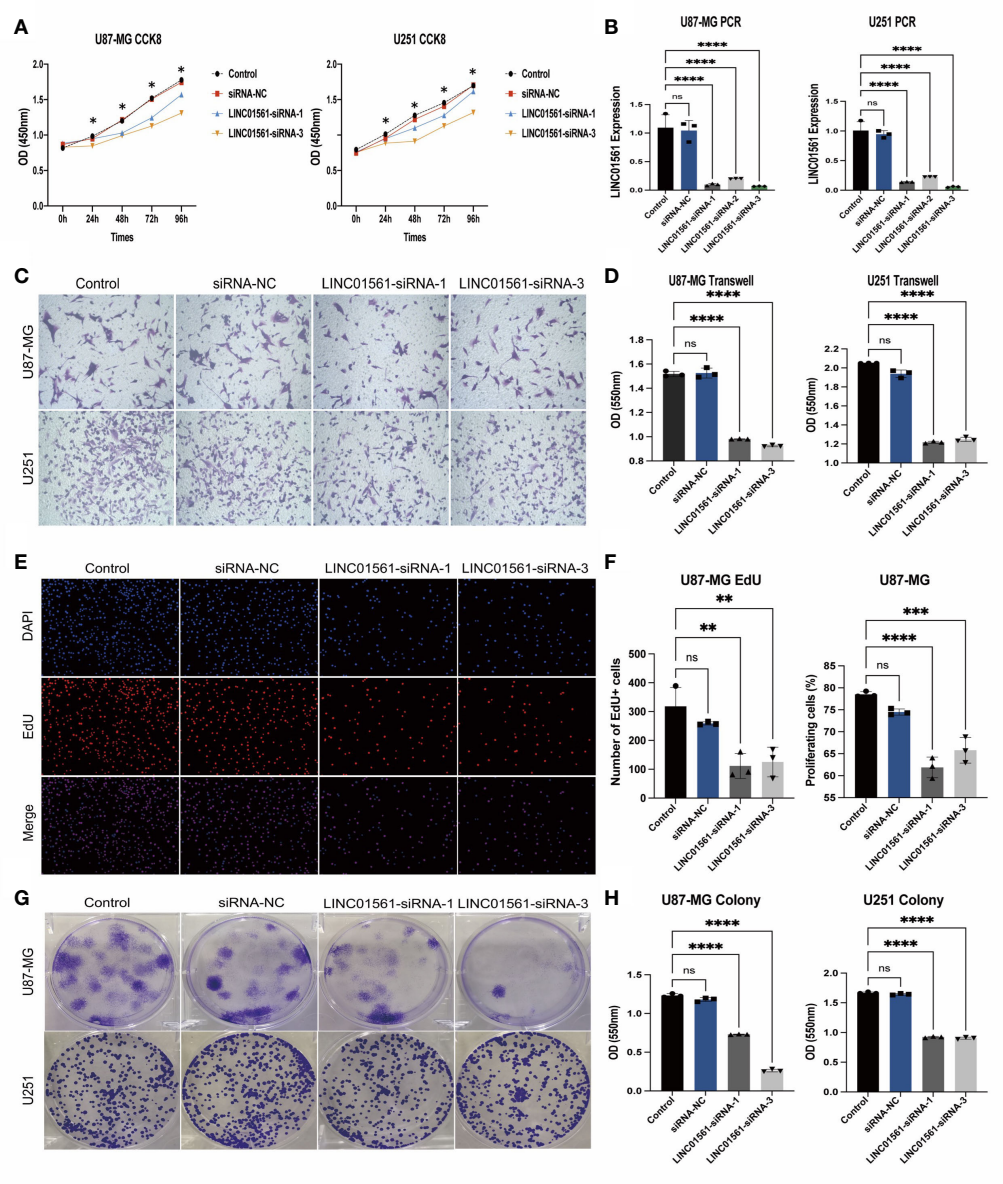
**(A)** Expression results of the CCK-8 assay of the silencing LINC01561 at different time points: 24, 48, 72, and 96h. **(B)** Relative expression levels of LINC01561 in control, siRNA-NC, and LINC01561-siRNA groups. **(C, D)** The results of Transwell assay in the control, siRNA-NC, and LINC01561-siRNA groups. **(E, F)** EdU assay showing the proliferation cells (U87-MG); EdU (red) and DAPI (blue) were stained. **(G, H)** Results of the colony-forming assay in LINC01561 expression. *p < 0.05, **p < 0.01, ***p < 0.001, ****p < 0.0001, ns p > 0.05.

To know the role of LINC01561 on glioma, we further conducted the effects of the increased level of LINC01561 in glioma cell lines (U251 and U87-MG) in *in vitro* experiments. The SiRNA technique was conducted to interfere with the mRNA expression of LINC01561 successfully, and it was confirmed by RT-PCR. CCK‐8 assay results showed inhibited cell viability after silencing LINC01561 expression. To further know the effect of LINC01561, a colony-forming assay was also conducted to suggest that the viability of glioma cells was significantly inhibited after LINC01561 was knocked down by siRNA. To further detect whether LINC01561 affects the metastasis of glioma, Transwell experiments were performed to assess the migration and invasion of glioma cell. These results demonstrated that siRNA-mediated LINC01561 silencing suppresses the invasion of glioma. Furthermore, the EdU proliferation assay indicated that inhibition of LINC01561 expression can lead to a significantly decreased EdU‐positive rate of glioma cells (**Figures 8**, **S4**).

## 4 Discussion

Glioma accounts for 80% of all malignant brain tumors (25) and is one of the most frequently occurring malignant central nervous system tumors in adults with a highly invasive and a poor prognosis (26). Currently, the prognosis of glioma patients was judged according to the criteria of the WHO classification in clinic (27). The development of molecular typing in gliomas has progressed rapidly based on the development of sequencing technology and bioinformatic technologies in recent years. Surgery is often unable to remove or completely resect the tumor because it is characterized by a highly invasive potential ability due to the aggressive growth of glioma and peripheral brain tissue, and residual tumor cells are difficult to be treated by radiation and chemotherapy thoroughly (28, 29). New targets for therapeutic approaches are significant for improving the prognosis of patients with glioma. Altered cellular metabolism is considered a hallmark of glioma cell biology (30, 31). Carbohydrates, lipids, and amino acids utilized by glioma cells and their initiating cells in the hypoxic lesions have important roles in the tumor microenvironment (30). Previous accumulating research mainly focused on lipid metabolism and glucose metabolism in glioma (32). Currently, a new focus on the amino acid metabolism of glioma is considered to play a major role in the glioma (33). In comparison with normal glioma tissues, glioma tissues exhibited higher expression levels of amino acid. LncRNAs have been reported to be involved in many cellular processes, which have been found abnormally expressed in many cancers (34). However, detailed analyses of lncRNAs and amino acid metabolism in glioma have not been reported previously; moreover, the role of amino acid metabolism remains unclear in glioma.

In our study, eight amino-related lncRNAs associated with glioma prognosis by bioinformatics analysis were found, namely, AL357060.1, HOXA-AS3, LINC01561, Z95115.1, AL353796.1, LEF1-AS1, AC005224.3, and TMEM220-AS1, which had significant differences in the expression in normal brain and glioma tissue. Therefore, an accurate predict prognostic lncRNA signature of patients was established. Of note, the amino acid metabolism-related risk signature identified in our study is still considered to be an independent prognostic factor after adjusting to the clinical and molecular features. The metabolic conditions of amino acid metabolism hold great potential and accurate predictive power for clinicopathological features. Together, combining the risk signature and other constraints can better predict a prognosis in patients with glioma. Moreover, in terms of further investigating the lncRNA signature, some researchers make an innovative methodological contribution, like having someone develop a new system assay for investigating associations on patterns of gene expression (35).

Long non-coding RNAs have been reported as new potentially promising therapeutic targets involved in multiple cancers (36). HOXA-AS3 has been reported to be upregulated in human pulmonary artery smooth muscle cells in the presence of hypoxia (5). HOXA-AS3 can regulate chemoresistance in several types of cancer (37). The expression of HOXA-AS3 increased in glioma patient samples and glioma cell lines (38). LINC01561, a newly identified tumor-related lncRNA, has critical regulatory roles in several tumors including non-small-cell lung carcinoma (39), lung squamous cell carcinoma (40), and breast cancer (41). However, no reports have shown the involvement of LINC01561 in glioma and its underlying mechanisms remain unknown. TMEM220-AS1 was identified as a new prognostic lncRNA biomarker in hepatocellular carcinoma. Downregulated lncRNA TMEM220-AS1 was associated with poorer prognosis in hepatocellular carcinoma (42). LEF1-AS1 is an lncRNA whose expression was significantly upregulated in glioma tissues and cell lines. The knockdown of LEF1-AS1 represses cell proliferation and suppressed tumor growth while activating apoptosis in glioma *via* the downregulated LEF1-AS1/miR-489-3p/HIGD1A axis (43). AL357060.1 has been reported as a new potential prognostic biomarker for hepatocellular carcinoma (44), but there is no report of its role in glioma. Low-risk patients have significantly longer progression-free and overall survival compared with the high-risk group.

Our study provided new insights into biological functions in the above signature lncRNAs, which participate in the amino acid metabolism of glioma. These lncRNAs mainly regulate amino acid biosynthesis, modification, catabolism, and translation. Amino acids (AAs) are essential for the function and survival of the cell. Pathways of glucose, amino acids, and fatty acid metabolism are involved in glioma reprogramming. Recently, great progress has been made in single-cell analysis (45), which is being generally used in terms of tumor study (33). Some scholars have developed a novel approach of the single-cell multi-omics co-regulatory algorithm to broaden our understanding of the underlying mechanisms in complex tumor (46). There is currently a lack of high-quality single-cell gene expression data for glioma. Our team is currently working with single-cell sequencing of glioma and will use this method as a reference for future research.

In the case of metabolic reprogramming of glioma, carbohydrates, lipids, and amino acids were efficiently utilized in glioma cells with hypoxic lesions. This ensures adequate energy to improve glioma rapid growth and migration while altering the role of cell features and its microenvironment (30). The tumor microenvironment holds an indispensable role in tumor metastasis. In recent years, the role of the immune microenvironment has been the subject of intensive research in gliomas (47). Immunotherapy, tumor microenvironment, and a combination of several efficacious methods have piqued ever-increasing interest (48). They include NK and T-cell dysfunction, T-cell and myeloid-derived suppressor cell expansion, immunosuppressive glioma cell surface factors and cytokines, and tumor microenvironment hypoxia. Gliomas have created a profoundly immunosuppressive environment, both topical and systemic within the tumor (49). Immunosuppressive tumor microenvironment reprogramming improved antitumor effectiveness (27). Moreover, tumor microenvironment-targeted therapies for gliomas may be a comprehensive source of the immune landscape and offers insights into possible strategies to overcome tumor with practical significance. MAT2A is an essential amino acid, and a high expression of MAT2A or an inhibitor of MAT2A can reduce the proliferation of glioma cells. Histone methylation is promoted by MAT2A, and cells can be prompted to proliferate in a methionine-restricted environment, which is associated with the progression of glioma (50). Compared with normal cells, rapidly proliferating tumor cells need higher demand for proteinogenic amino acids, and the bioavailability of the use of amino acids is affected largely in human proteomes (51). There were tight associations between serine, glycine, and other non-essential amino acids and tumorigenesis and tumor development. Much research has been devoted to molecular biomarkers that may contribute to the diagnosis or treatment of glioma, which revealed a series of biomarkers associated with the prognosis of glioma. Initiation and development of glioma involve metabolic alterations and genetic and epigenetic alterations on the molecular level, including lncRNA expression. However, the effects of molecular biological mechanisms on amino acid metabolism are poorly understood. Therefore, amino-related lncRNAs found in our study provided new insights to be considered as new genetic biomarkers for the prognosis and treatment of glioma.

As mentioned above, LINC01561 is a novel identified tumor-related lncRNA associated with the progression and poor prognosis of glioma. However, to date, no reports have investigated the involvement of LINC01561 in glioma and its underlying mechanisms remain unknown. We, therefore, have selected LINC01561 for further study of verification analysis.

Our study revealed a negative correlation between LINC01561 expression and the prognosis of glioma patients, indicating that LINC01561 can serve as a reliable predictor of glioma prognosis. It was somewhat that lncRNA exhibited the highest prognostic value. Moreover, LINC01561 was involved in regulating the immune microenvironment of gliomas, which plays a crucial role in tumor initiation and prognosis (25).

Based on our *in vitro* experiments, we found that siRNA-mediated inhibition of LINC01561 suppressed the proliferation of U87-MG and U251 cells, as confirmed by the CCK-8, colony formation assays, and EdU assays. Transwell experiments revealed that silencing LINC01561 inhibits glioma cell invasion, indicating that downregulation of LINC01561 significantly hindered glioma progression. Taken together, these results demonstrate that LINC01561 is a novel identified tumor-related lncRNA playing an important regulatory role in glioma, potentially involved in multiple biological processes.

## 5 Conclusion

In this study, our bioinformatics analysis identified eight novel amino acid-related lncRNAs associated with glioma patient survival and established an lncRNA signature for prognostic and therapeutic prediction. These findings suggest that the identified lncRNAs may play important roles in glioma, highlighting the importance of amino acid metabolism in glioma and the need for further research. Although our study identified and selected several key lncRNAs, such as LINC01561, more detailed studies are still required to elucidate their roles in regulating amino acid metabolism and their specific mechanisms of action. Therefore, further studies are warranted to advance our understanding of glioma and develop more effective management strategies for this disease.

## Data availability statement

The raw data supporting the conclusions of this article will be made available by the authors, without undue reservation.

## Author contributions

Conception and design: QL and ZX. Foundation support: QL and ZX. Experiment: LP, and BY. Acquisition and analysis of data: KL, LP, ZX, and QL. Interpretation of data: ZX and QL. Drafting the manuscript and revising for submission quality: QL, BY, KL, LP, and ZX. Study supervision: LP and ZX. All authors contributed to the article and approved the submitted version.

## Supplementary material

Supplementary Figure 1
**(A-F)** The difference in K-M plots of the OS and PFI between high- and low-risk groups in the dataset of TCGA. **(A-F)** The difference in K-M plots of OS between high- and low-risk groups in the dataset of CGGA.

Supplementary Figure 2 The survival status of different subgroups of gliomas. **(A)** Age, **(B)** Cancer type, **(C)** Gender, **(D)** Grade, **(E)** IDH type, **(F)** MGMT status, **(G)** 1p/19q status, **(H)**Cancer subtype, **(I)** Combined cancer subtype.

Supplementary Figure 3 Consensus Cluster analyses of the 8 lncRNA expressions in the CCCA dataset. **(A)** Clustering stability for k=2 to 10. **(B)** The incremental change under the CDF curve area. **(C)** The consensus clustering of gliomas was clustered into two clusters in principal component analysis (PCA). **(D-F)** The difference in K-M plots of the OS between the cluster 1 and cluster 2 in CGGA dataset.

Supplementary Figure 4
**(A)** The results of Transwell assay in control, siRNA-NC, and LINC01561-siRNA groups in U251 cells. **(B, C)** The EdU assay showing the U251 cells proliferation; EdU (red) and DAPI (blue) were stained.

Supplementary Table 1
*In Vitro* Cell Experiments supplementary materials
