# Supplementary figures and images for: A novel prognostic related lncRNA signature associated with amino acid metabolism in glioma
Source: Front Immunol. 2023 Apr 11;14:1014378. doi: 10.3389/fimmu.2023.1014378 (PMC10126287; doi:10.3389/fimmu.2023.1014378)

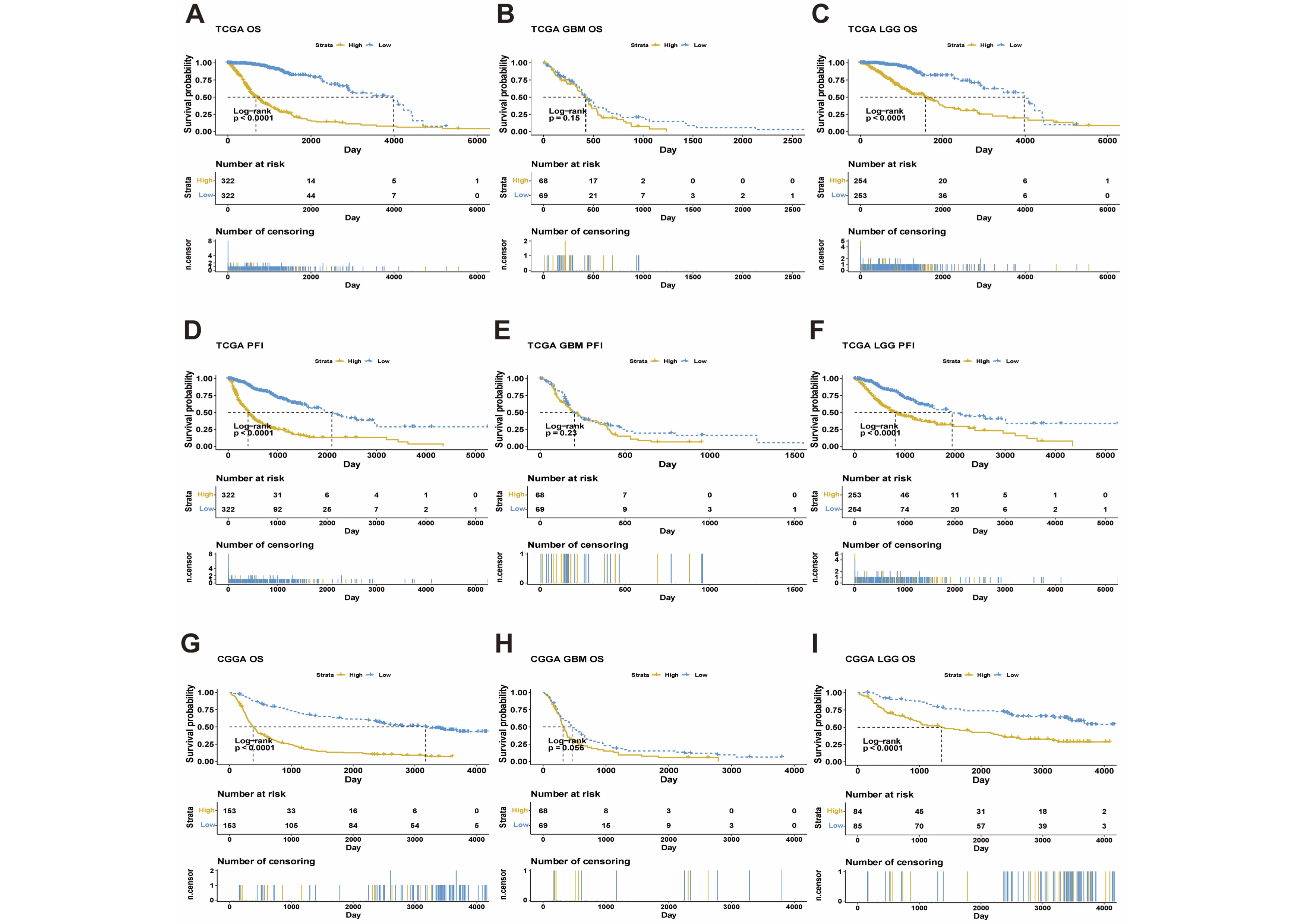

Supplement: Supplementary Figure 1 — (A-F) The difference in K-M plots of the OS and PFI between high- and low-risk groups in the dataset of TCGA. (A-F) The difference in K-M plots of OS between high- and low-risk groups in the dataset of CGGA. [file Image_1.jpeg]

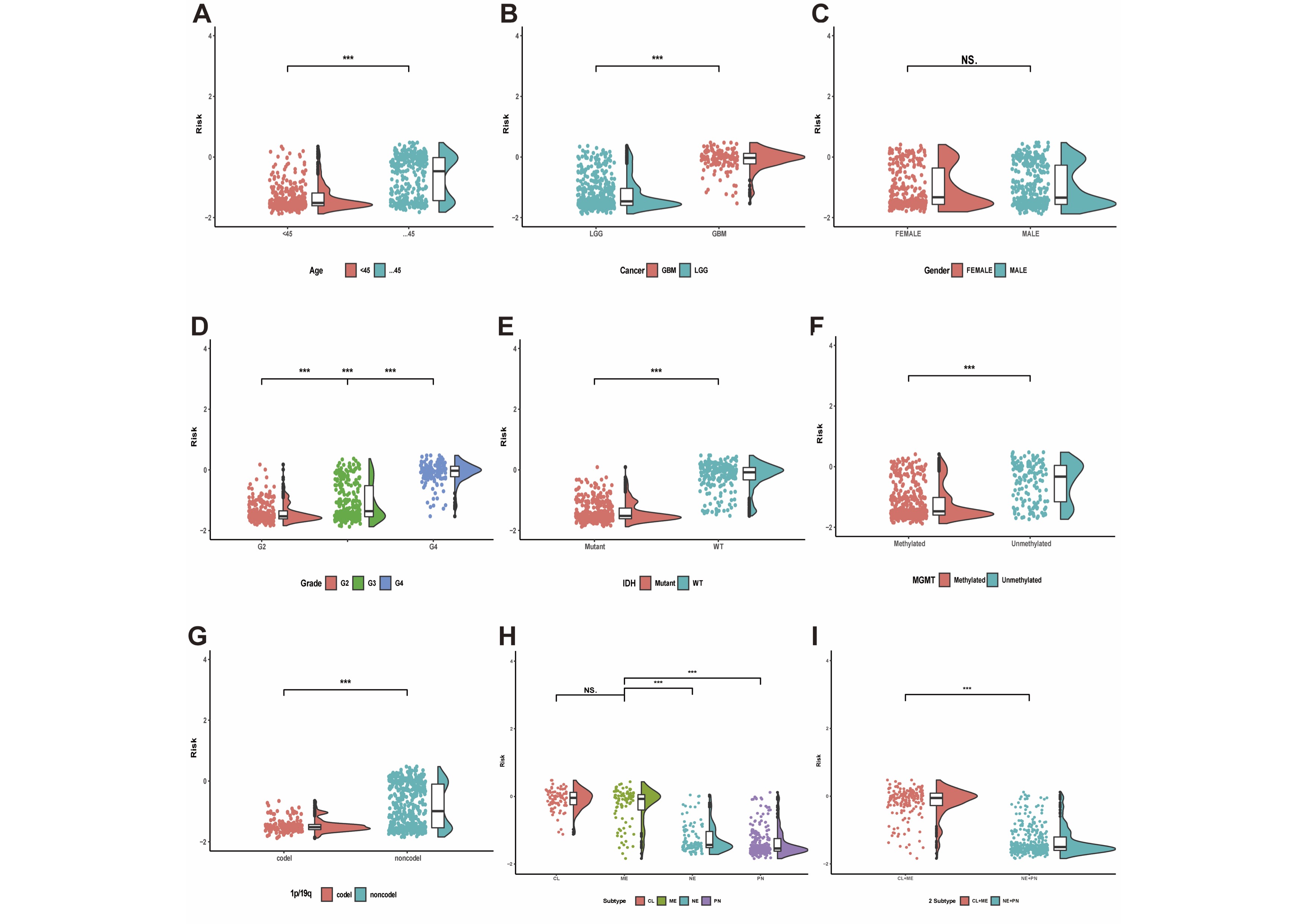

Supplement: Supplementary Figure 2 — The survival status of different subgroups of gliomas. (A) Age, (B) Cancer type, (C) Gender, (D) Grade, (E) IDH type, (F) MGMT status, (G) 1p/19q status, (H)Cancer subtype, (I) Combined cancer subtype. [file Image_2.jpeg]

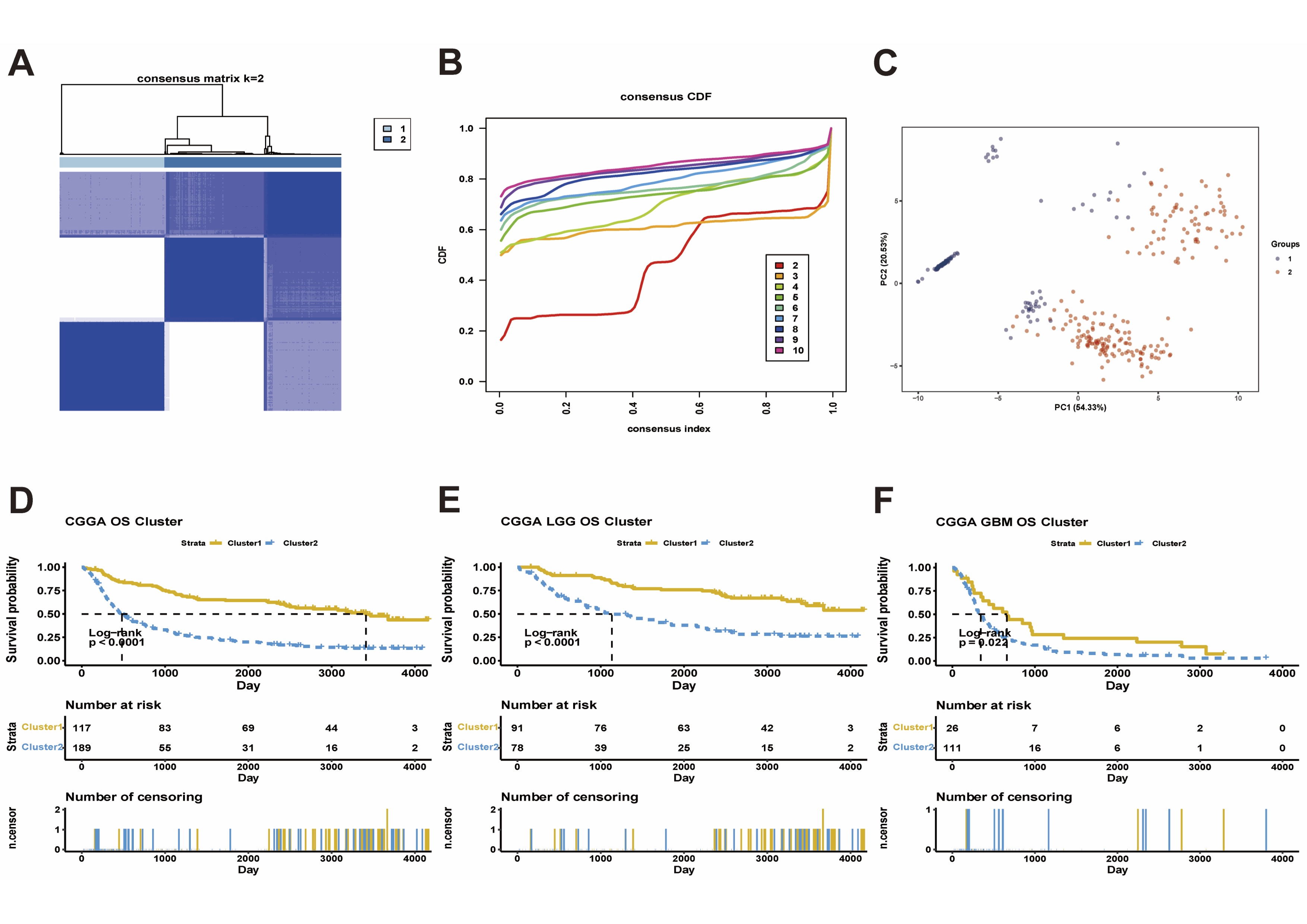

Supplement: Supplementary Figure 3 — Consensus Cluster analyses of the 8 lncRNA expressions in the CCCA dataset. (A) Clustering stability for k=2 to 10. (B) The incremental change under the CDF curve area. (C) The consensus clustering of gliomas was clustered into two clusters in principal component analysis (PCA). (D-F) The difference in K-M plots of the OS between the cluster 1 and cluster 2 in CGGA dataset. [file Image_3.jpeg]

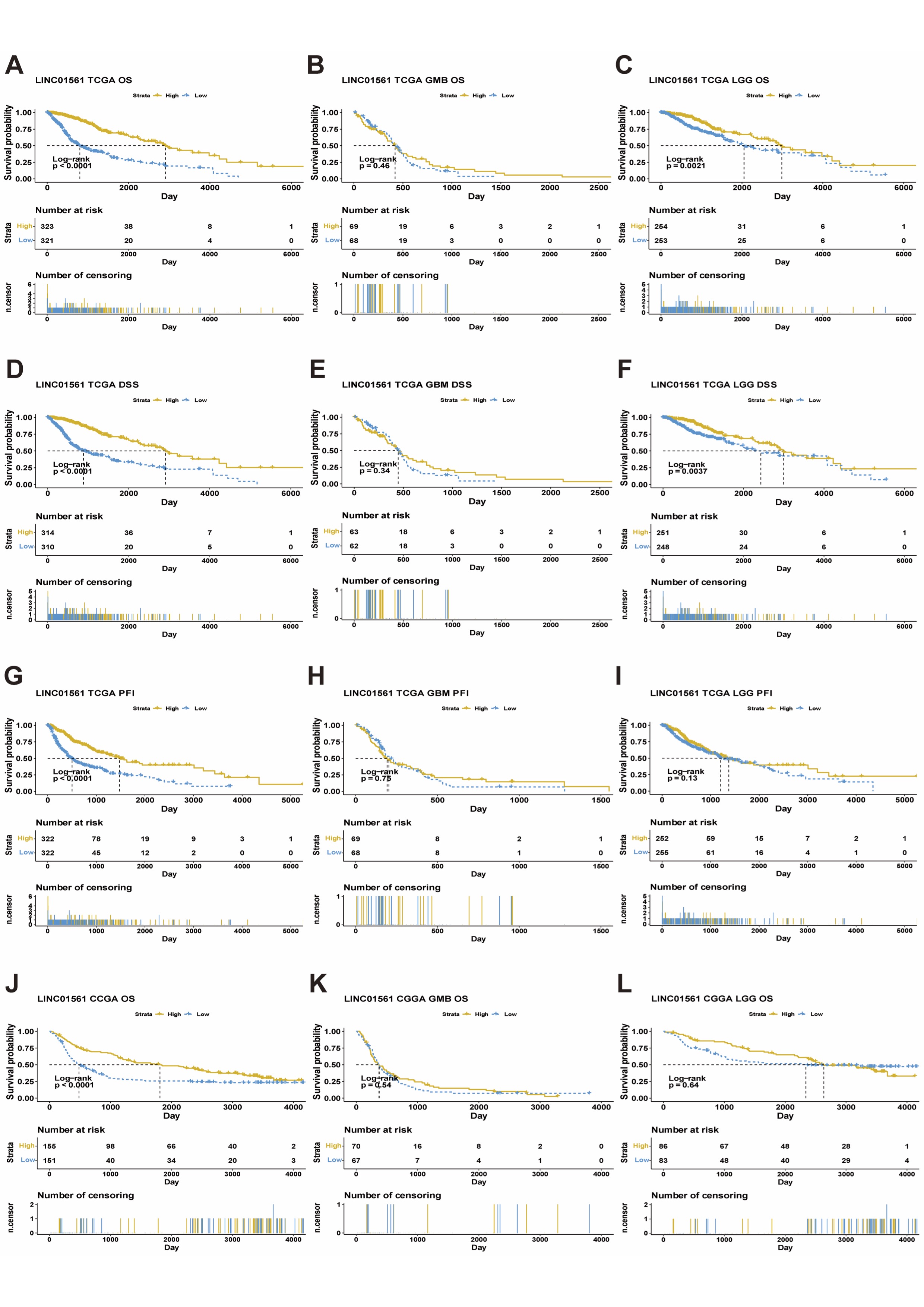

Supplement: Supplementary Figure 4 — (A) The results of Transwell assay in control, siRNA-NC, and LINC01561-siRNA groups in U251 cells. (B, C) The EdU assay showing the U251 cells proliferation; EdU (red) and DAPI (blue) were stained. [file Image_4.jpeg]

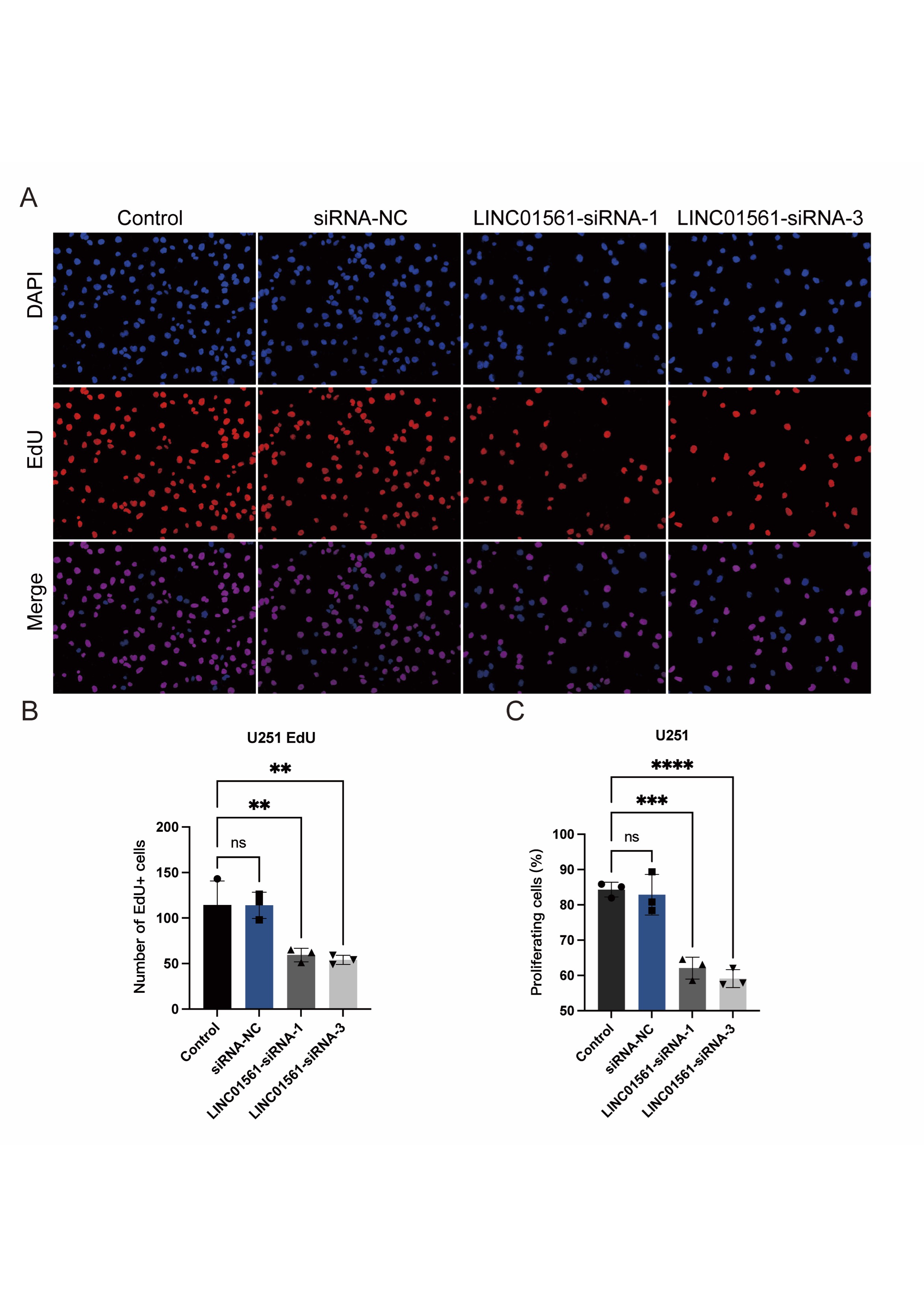

Supplement: Supplementary Table 1 — In Vitro Cell Experiments supplementary materials [file Image_5.jpeg]
