# Supplementary material for: A novel prognostic related lncRNA signature associated with amino acid metabolism in glioma
Source: Front Immunol. 2023 Apr 11;14:1014378. doi: 10.3389/fimmu.2023.1014378 (PMC10126287; doi:10.3389/fimmu.2023.1014378)
Supplement: Supplementary file 6 [file Table_1.docx]

**Table S1. In Vitro Cell Experiments supplementary materials**

**Cell counting kit-8 (CCK-8) assay to assess cell viability:**

1. Logarithmic-phase cells were digested and counted, and then seeded at a density of 5,000 cells per well in a 96-well plate with 100 μL of the medium. Three replicates were set for each group.
2. After adhering to the wall, the cells were treated for a certain amount of time and then 10 μL of CCK8 solution was added to each well. The CCK8 solution was prepared by diluting the fully supplemented medium with CCK8. After removing the medium with drugs, 100 μL of CCK8-containing medium was added to each well.
3. The cells were then incubated for an additional 4 hours at 37℃ and 5% CO2, and the absorbance at 450 nm was measured using a Bio-Tek microplate reader.

**Transwell assay to measure cell migration:**

1. Add 500 μl of 10% FBS serum complete medium to the lower chamber.
2. Digest cells with trypsin to obtain single cells, then resuspend cells to a concentration of 2×10^6 cells/ml and add 100 μl of cells to each well.
3. Incubate in a 37°C incubator for 48 hours.
4. Take out the upper chamber and wash it with PBS to remove non-migrated cells, then dry the upper chamber cells with a cotton ball.
5. Fix with 4% paraformaldehyde for 20 minutes, then remove the membrane.
6. Stain with 0.1% crystal violet for 5 minutes, and wash with water 5 times.
7. Place the membrane on a glass slide and take pictures under a microscope.
8. Observe the cells on the outer surface of the upper chamber and take three fields of view for each.
9. Decolorize the lower chamber with 10% acetic acid and measure the absorbance (OD) at 550 nm using a Microplate Reader. Repeat each group three times.

**Cell colony formation assay to assess colony forming ability:**

1. Take cells from each group in the exponential growth phase, digest them with 0.25% trypsin, and blow them into single cells. Suspend the cells in a complete culture medium containing 10% fetal bovine serum and set aside.
2. In a 6-well plate containing 1mL of recovery-to-room-temperature culture medium per well, seed 200 cells per group of suspended cells and gently rotate to evenly disperse the cells. Place the plate in a cell culture incubator at 37°C, 5% CO2, and saturated humidity, and culture for 2-3 weeks with periodic medium changes.
3. Observe regularly and terminate the culture when visible clones appear in the culture dish.
4. Discard the culture medium, rinse with PBS solution twice, and add 1mL of 4% paraformaldehyde solution per well to fix the cells for 15 minutes.
5. Remove the fixative solution and add 1mL of working staining solution at room temperature for 30 minutes.
6. Slowly rinse away the staining solution with running water and air dry.
7. Take photos of the cells using a smartphone camera.
8. To measure the OD value, remove the 6-well plate, soak it in 1mL of 10% acetic acid solution to remove the color, and measure the absorbance (OD) value at 550nm with an enzyme-linked immunosorbent assay (ELISA) reader. Repeat the measurement three times for accuracy.

**The 5-ethynyl-2'-deoxyuridine (EdU) assay to assess cell proliferation:**

1. EdU labeling

1.1 Dilute EdU solution (reagent A) in cell culture medium at a ratio of 1000:1 to prepare 50μM EdU medium.

1.2 Add 100μL of 50μM EdU medium to each well and incubate overnight, discard the medium.

1.3 Wash the cells with PBS 1-2 times, each time for 5 minutes.

1. Cell fixation

2.1 Add 50μL of cell fixation solution (4% paraformaldehyde) to each well and incubate at room temperature for 30 minutes, discard the fixation solution.

2.2 Add 50μL of 2 mg/mL glycine to each well, incubate on a shaker for 5 minutes for decolorization and discard the glycine solution.

2.3 Add 100μL of PBS to each well, incubate on a shaker for 5 minutes for decolorization, and discard the PBS.

2.4 Add 100μL of permeabilization solution, incubate on a shaker for 10 minutes, and wash with PBS for 5 minutes.

1. Apollo staining

3.1 Add 100μL of 1x Apollo® staining reaction solution to each well, incubate on a shaker in the dark at room temperature for 30 minutes, and discard the staining reaction solution. 3.2 Wash with permeabilization solution 2-3 times, each time for 10 minutes, and discard the permeabilization solution.

3.3 Wash with methanol 1-2 times, each time for 5 minutes, and wash with PBS 1 time, each time for 5 minutes.

1. DNA staining

4.1 Dilute reagent F in deionized water at a ratio of 100:1 to prepare 1x Hoechst33342 reaction solution and store it in the dark.

4.2 Add 100μL of 1x Hoechst 33342 reaction solution to each well, incubate on a shaker in the dark at room temperature for 30 minutes, and discard the staining reaction solution. 4.3 Wash with PBS 1-3 times, each time for 100μL.

1. Image acquisition and analysis

Observe immediately after staining completion.
